# A Novel Smart Shoe Instrumented with Sensors for Quantifying Foot Placement and Clearance during Stair Negotiation

**DOI:** 10.3390/s23249638

**Published:** 2023-12-05

**Authors:** Malarvizhi Ram, Vasilios Baltzopoulos, Andy Shaw, Constantinos N. Maganaris, Jeff Cullen, Thomas O’Brien, Patryk Kot

**Affiliations:** 1 Research to Improve Stair Climbing Safety (RISCS), Faculty of Science, School of Sport and Exercise Sciences, Liverpool John Moores University, Byrom Street, Liverpool L3 3AF, UK; v.baltzopoulos@ljmu.ac.uk (V.B.); c.maganaris@ljmu.ac.uk (C.N.M.); 2 Faculty of Engineering and Technology, Liverpool John Moores University, Byrom Street, Liverpool L3 3AF, UK

**Keywords:** foot contact length, wearable sensor insole, force sensitive resistor, fall risk prediction

## Abstract

Trips and slips are significant causal perturbations leading to falls on stairs, especially in older people. The risk of a trip caused by a toe or heel catch on the step edge increases when clearance is small and variable between steps. The risk of a slip increases if the proportion of the foot area in contact with the step is reduced and variable between steps. To assess fall risk, these measurements are typically taken in a gait lab using motion-capture optoelectronic systems. The aim of this work was to develop a novel smart shoe equipped with sensors to measure foot placement and foot clearance on stairs in real homes. To validate the smart shoe as a tool for estimating stair fall risk, twenty-five older adults’ sensor-based measurements were compared against foot placement and clearance measurements taken in an experimental staircase in the lab using correlations and Bland–Altman agreement techniques. The results showed that there was a good agreement and a strong positive linear correlation for foot placement (r = 0.878, *p* < 0.000) and foot clearance (r = 0.967, *p* < 0.000) between sensor and motion analysis, offering promise for advancing the current prototype into a measurement tool for fall risk in real-life staircases.

## 1. Introduction

Falls are common, but older people are the most vulnerable group, with falls leading to injuries and death if serious. Approximately 1 in 3 older people aged over 65 and living independently in a home environment experience at least one fall a year [1]. Although not all falls cause serious injuries, some falls lead to bone fractures and mobility issues that reduce confidence. When older people lose their confidence, they become withdrawn and dependent, which has several serious consequences [2].

One of the most dangerous and challenging tasks for older individuals is negotiating stairs, which frequently results in falls, with 7–35% of all falls occurring on stairs [3]. Compared to level walking, fewer falls occur on stairs; however, these stair falls are the second most common reason for fatal accidents [4] and cost the National Health Service (NHS) in the UK approximately 2.3 billion EUR annually [5].

Older people’s fall risk parameters are normally examined on instrumented stairs in a gait or biomechanics laboratory setting using motion capture equipment and force plates [6]. These motion capture systems produce accurately quantified results for body movement kinematics examined in the laboratory. Based on such laboratory-based studies [6,7,8], it was determined that an overstep-induced fall is one of the most important reasons for stair falls during descent. If the amount of the foot in contact with the step decreases, there is an increased chance of slipping [7]. In addition, as the variability of the foot in contact with the step increases, the chance for slip increases, as this may suggest an inability to position the foot consistently and securely on the step [7]. In addition, the risk of tripping during an ascent is generally related to the distance between the forefoot and the step edge [9], which is called foot clearance. The risk of tripping increases when the vertical distance of the foot to the edge of the step is reduced [9]. In addition, as the variability in foot clearance increases, the risk of tripping increases, as this may indicate that a person cannot maintain a safe and consistent foot trajectory [10].

However, these laboratory staircase biomechanics procedures are costly, require dedicated motion laboratory space, and use cumbersome equipment that might interfere with the movement. Moreover, several environmental differences make it practically impossible to accurately simulate an individual’s home stair scenario in the laboratory. These include ecological differences between houses, such as the shape of the staircase, the dimensions of the steps, the material used to construct and cover the steps, and the lighting of the stairwell. Therefore, it is not feasible to systematically study and document what causes falls on staircases in real houses where people live by only examining stair biomechanics in laboratory settings.

So, there is an urgent need for a practical sensor-based system to directly measure key fall risk variables, such as foot placement and foot clearance in home environments. An instrumented insole offers several advantages when compared with force platforms or floor-mounted systems for such measurements in real homes. Participants can wear the instrumented insole while negotiating stairs in their own homes. The insole can monitor multiple steps as opposed to a fixed force plate and can also be used in the laboratory or clinic, home, and outdoor environments, because of wireless communications development. 

The recent advances in wearable sensor technology enable us to pursue staircase research with small and inexpensive sensors. Currently, there is no dedicated sensor insole available to calculate foot placement on the stairs. However, there are some sensor insoles available to measure plantar pressure during gait analysis. For example, the main commercially available in-shoe measurement systems are from F-Scan Systems and Pedar, but these systems are costly. There are other sensor insoles for specific applications. For example, an instrumented system was developed by [11] based on miniature electronic circuits that collect and transmit data from force-sensing resistors for gait monitoring of Parkinsonian patients. Force sensor-based insoles are also used to calculate foot force distribution during level walking, and this allows examining force changes under localized regions and extracting the foot’s orientation and position during gait [12].

In addition, many other instrumented insole designs were developed with several sensors to measure different kinematic and kinetic parameters based on specific research [13,14] requirements. However, all these customs, or instrumented sensor insoles, did not target or cannot be used for measuring foot placement (foot overhang). 

A shoe-integrated system for direct foot clearance measurement is the most unexplored topic in stair gait analysis and biomechanics research. An accelerometer sensor has been used to calculate foot clearance in level walking. However, accelerometer data provides unreliable results because of drifts and errors [15]. Sensing minimum foot clearance (MFC) using only accelerometer measurements on uneven, bumpy surfaces or during stair descent or ascent is problematic. It does not directly measure foot clearance but instead derives it indirectly using acceleration data, so it is unreliable. 

MIT Media Laboratory has developed an electric field sensing technique and proved that this technique can calculate foot clearance during level walking [16]. The drawback of this system is that it can only measure clearances up to 5 cm [17]. An ultrasonic sensor has been used to calculate foot clearance [18], but the problem with this system is that the ultrasonic sensor is too big to fit in the shoe, which might affect the participants’ walking. The studies mentioned above focus on level walking foot clearance, so there is an urgent need for a simple, low-cost sensor to reliably detect foot clearance on the stairs. 

There are different approaches available to track human motion, with the main ones being optical motion systems (such as VICON or Optotrak) and commercial IMU sensors such as MTw (Xsens). However, the most widely used and accepted system to track stair climbing motion is optical motion systems because they have high accuracy when operating in controlled environments. The VICON optical motion system was used in our lab, so this was chosen to compare against the sensor-based results. There are several studies available for sensor-based fall risk detection on ground level, particularly [19] presents a comprehensive survey to discuss the development and current status of various aspects of sensor-based fall risk assessment on ground level. Another paper compares fall risk using FSR on level ground and provides a comparable chart [20]. However, there are no studies available for stair fall risk detection with sensors particularly focusing on foot clearance and foot contact length, so there is an urgent need for a simple, low-cost sensor to reliably detect foot clearance on the stairs. 

The aim of this research work was to develop a novel smart shoe instrumented with sensors for quantifying foot placement and clearance to identify stair fall risk in a typical real-life living environment. The simple wearable sensors and insole were developed to investigate foot placement and clearance by collecting a vast quantity of data in different domestic staircases in real houses and living environments that are impossible to collect with the current laboratory-based motion analysis systems.

## 2. Materials and Methods

### 2.1. System Design

**Figure 1:**
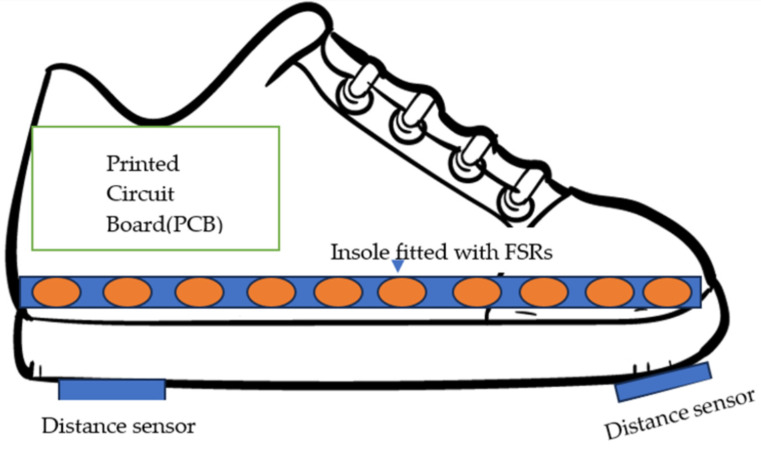
Instrumented sensor shoe overall diagram with insole.

Figure 1 shows the instrumented sensor shoe’s circuit diagram. An instrumented sensor shoe was developed with two distance sensors fitted under the shoe to calculate foot clearance. Force-sensitive resistors were used to calculate foot contact length, which was fitted in the insole. 

**Figure 2:**
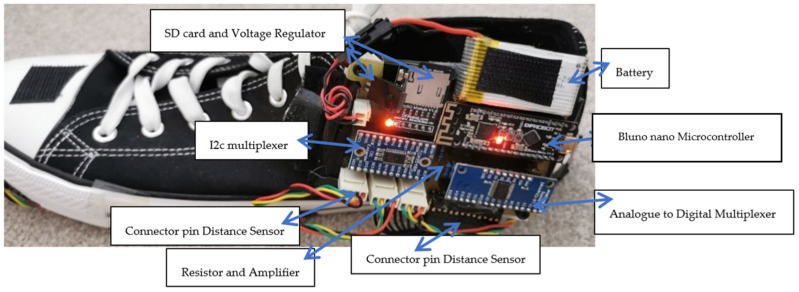
Instrumented sensor shoe with PCB module.

Figure 2 shows the instrumented sensor shoe with a printed circuit board (PCB) module. A printed circuit board (PCB) is vital in holding the entire instrumented shoe’s sensors and other hardware together. The PCB module contained the Bluno Nano microcontroller with Bluetooth (BLE), making the instrumented shoe into a wireless system. The PCB module contains additional components, which are an analog multiplexer, resistors, an amplifier for the insole, a voltage controller, and a connection for the inputs from the power supply.

The Atmega 328 microcontroller chip (Atmel, San Jose, VA, USA) was used in the Bluno Nano board. Bluno Nano integrates a TI CC2540 BT 4.0 chip (Texas Instruments, Dallas, TX, USA) with the Arduino UNO development board (DFROBOT 2015, Shanghai, China). It allows wireless programming via Bluetooth Low Energy (BLE) and supports the AT command to configure the BLE. The Bluno Nano board contains an integrated Bluetooth module that directly transmits any data written over a ‘Bluetooth Low Energy (BLE)’ connection to its serial port. An I2c multiplexer was used to collect data across two sensors that were concurrently operating I2c channels. Spark Fun’s Analog to Digital Multiplexer CD74HC4067 (Spark Fun, Boulder, CO, USA) breakout board was used to multiplex FSR sensors. The 7.4 V rechargeable batteries (abaos, Shenzhen, China) were used to provide the required voltage to the whole design for smooth operation. Voltage regulators were used in this design to regulate and maintain the input voltage at a constant level. Data storage is a sub-system used by the instrumented sensor shoe to store the collected data for further analysis. An individual micro-SD card for each shoe was used in this model to record the real-time data and store the collected data. The data was stored as CSV files by using LabVIEW 13.0.1 software (National Instruments, Austin, TX, USA).

After designing the PCB, two separate PCBs were printed for both left and right feet to instrument both shoes and obtain data from both sides at the same time. Figure 2 shows the instrumented sensor shoe. The instrumented shoe comprised one PCB module and an instrumented insole. The PCB module was mounted to the shoe’s lateral side using a robust Velcro strap. The instrumented insole was placed inside the shoe, and we used shoe sizes 6 to 10 to accommodate women and men participants in our research.

### 2.2. Instrumented Insole Design to Measure Foot Contact Length

Force-sensitive resistors (FSR) were used to develop an instrumented insole to measure foot contact length. An FSR is a type of resistive sensor that experiences a decrease in electrical resistance when force is applied orthogonally to the sensor’s active area [21]. Though less accurate than a load cell, FSRs are generally inexpensive, and when manufactured from polymers, the typical thickness is on the order of 0.25 mm. We used the FSR, manufactured by Interlink Electronics (Camarillo, CA, USA) (FSR402 short), which has a circular sensing area with a diameter of 13 mm (Electronics 2010). Up to ten FSRs were placed underneath the foot from toe to heel, depending on shoe size, to calculate foot placement when the foot was in contact with the step during stair negotiation.

#### FSR Working Principle

**Figure 3:**
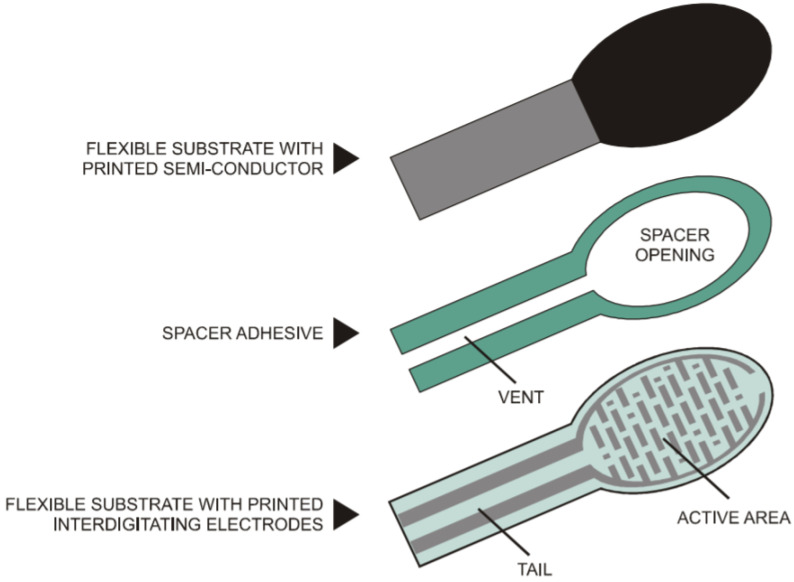
FSR composed of these three layers [21].

The FSR-402s are polymer-based sensors and consist of three layers. The lowest layer is a flexible substrate, which is coated with a printable semiconductor material. The middle layer is a spacer adhesive, with material only along the outline of the part, providing an open region at the device’s active area. The top layer is a flexible substrate printed with interdigitating electrodes and two printed leads connected to solder tabs. The active area of the sensor is the area containing the electrodes. FSR-402 uses a 0.13 mm layer of polyetherimide for the top and bottom layers, with a 0.15 mm layer of acrylic for the spacer. The transparent polyether sulfone has excellent temperature resistance, moderate chemical resistance, and good flexibility. On the other hand, polyetherimide is a semitransparent substrate with excellent temperature resistance, excellent chemical resistance, and limited flexibility (Figure 3).

The semiconductor material on the lowest layer of the FSR provides an electrical connection between the sets of interdigitating electrodes. The adhesive provides an air gap between the semiconductor and the electrodes when there is no force applied; this maintains the high sensor resistance. When force is applied across the active area, the electrodes are pressed into the semiconductor, reducing the resistance across the sensor. Table 1 shows the FSR parameters and specification [21].

**Figure 4:**
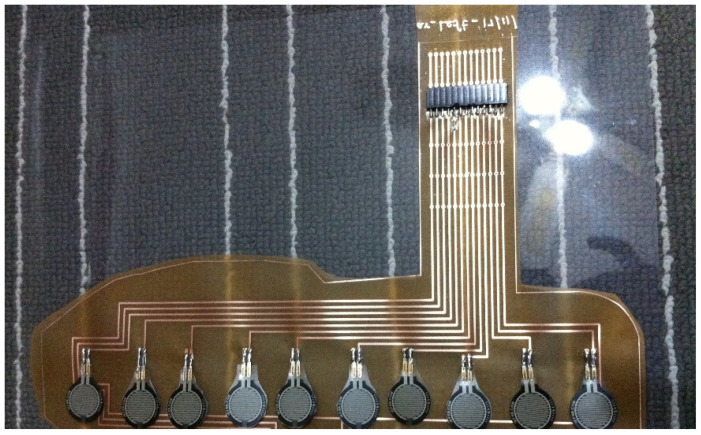
Insole design with FSRs.

The Eagle software (EAGLE 6.5.0 light) was used to design the instrumented insole, and flexible PCB lamination material was used to print the insole design. The advantage of using this flexible insole was that it removed wires inside the shoe. Figure 4 shows the insole design. The flexible PCB (Flex FR4) was just 0.127 mm thick, double-sided, from 1/2 oz, copper material, and it is ideal for making flexible circuits. After printing a flexible insole, FSRs were soldered onto it. The instrumented insole accommodates several FSR sensors connected to the PCB module via a connector pin.

The insole contained from 6 to 10 FSRs and provided a straightforward and convenient method to position the sensors in order at the plantar surface beneath the foot. The reason for placing the sensors in line was to calculate foot placement directly. This provides force readings only when the foot touches the steps during the stance phase, with no force readings during the swing phase or when the foot is not in contact with the stairs. 

A voltage divider circuit was used to measure changes in the resistance of the FSRs; the output voltage produced by this circuit is inversely proportional to the resistance of the FSR, which is inversely proportional to the applied force. A 5-volt power supply and a 10-kohm fixed resistor (R) were used to maximize the desired force sensitivity range and limit the current. This voltage divider circuit implementation produced a nonlinear output.

**Figure 5:**
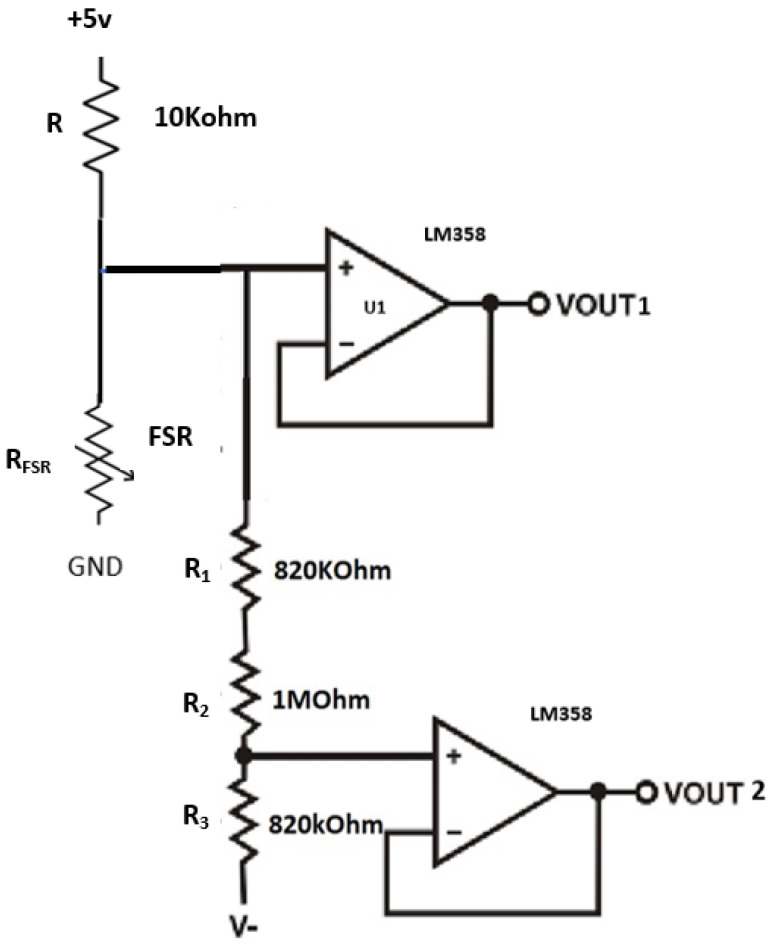
To overcome nonlinear output, a new voltage divider circuit was created with two operational amplifiers (Op-amp).

To solve this nonlinear problem, a new circuit was implemented with an additional FSR divider and a unity gain amplifier using ‘Op-amp LM-358’ (Onsemi, Suzhou, China) as shown in Figure 5. This offset-compensated ‘Op-amp LM-358’ offers high input impedance that has been recommended for use by Interlink Electronics (FSR integration guide). Two identical amplifiers were incorporated onto a single board, which does the parallel processing. The comparator can compare two voltages (reference voltage and the sensing voltage). The reference voltage is a set force threshold voltage (1.1 V) used to compare against the sensing voltage coming from the voltage divider. When the sensing voltage exceeds the reference voltage, the Op-amp turns on. The circuit output was divided into two parallels: vout-1 to work with 2/3-threshold force and vout-2 to work with 1/3-threshold force. This helps the FSR measure higher forces using vout-1 and lower forces using vout-2. The advantage of using this method is the accurate measurement of different levels of force, which are higher and lower. When the real force is applied in the FSR, vout-1 will produce the higher force readings; when a slight touch force is applied in the FSR, vout-2 will produce the lower force readings. So, for further analysis, higher force readings are used to check whether real force is applied or not. The Tinius Olseen Material Testing Machine (Horsham, PA, USA) was used for FSR calibration.

To avoid potential problems with body weight, FSRs were developed to measure light forces and higher forces based on some threshold. We were only interested if a sensor reads a force value above a threshold or not), only those values were used for further processing. We only got higher force readings when real forces were applied slight touching and tight shoes gave a reading around 0 N, which we did not use for further analysis. Detailed FSR calibration is available in the Supplementary Materials.

### 2.3. Instrumented Sensor Design to Measure Foot Clearance 

The instrumented sensor shoe design and foot clearance parameter calculation have been presented and tested in younger adults earlier [22]. In this paper, the same instrumented shoe was used to test older people. 

The VL6180X product is based on ST’s patented ‘Flight Sense™ technology’. This ground-breaking technology allows absolute distance to be measured independent of target reflectance [23]. Instead of estimating the distance by measuring the amount of light reflected from the object (which is significantly influenced by color and surface), the VL6180X precisely measures the time the light takes to travel to the nearest object and reflect the sensor (time-of-flight) [23]. Combining an IR emitter, a range sensor, and an ambient light sensor in a three-in-one, ready-to-use package, the VL6180X is easy to integrate. 

#### Working Principle

**Figure 6:**
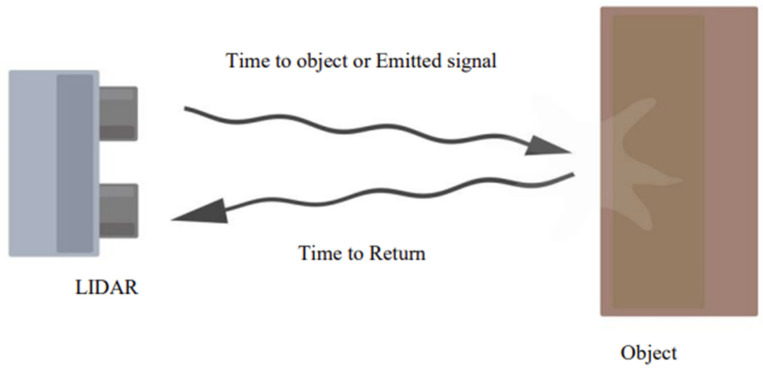
Distance sensor working principle.

The VL6180X uses ST’s ‘Flight Sense technology’ to measure how long it takes for emitted pulses of infrared laser light to be reflected to a detector from an object, making it essentially a short-range LIDAR (light detection and ranging) sensor. This ‘time-of-light (TOF)’ measurement enables it to accurately determine the absolute distance to a target with 1-mm resolution without the object’s reflectance influencing the measurement [23]. It can measure distances up to 250 mm. The microcontroller embedded in the ST devices handles all calculations and noise reduction. The reasons for choosing this sensor are low cost, small size, and accuracy. Figure 6 shows the TOF concept, multiplying the time frame and the speed of light in the air provides the distance.

Speed of light(c) = 299,792,458 m/s

Time(t) = (To object + Return)/2

Distance = Speed of light × Time

**Figure 7:**
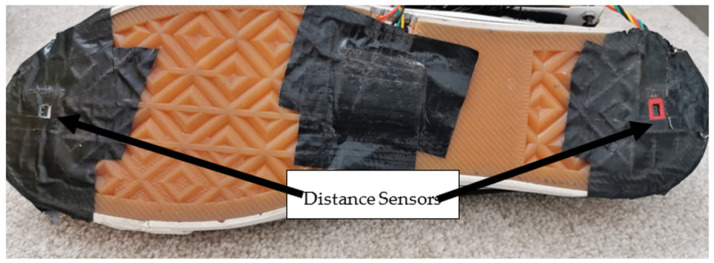
Distance sensors fitted underneath the shoe.

Table 2 shows the specification of the selected distance sensor for foot clearance [23]. A VL6180X (STMicroelectronics, Geneva, Switzerland) distance sensor was selected to measure foot clearance; this sensor was chosen due to its size, measurement range, and accuracy. This sensor contains a distance sensor and a light sensor. Two distance sensors were implemented to measure foot clearance. Two distance sensors were placed underneath the shoe’s sole, under the toes, and on the heel (Figure 7). The front sensor was used to calculate ascending vertical foot clearance, and the rear sensor was used for descending vertical foot clearance.

Foot clearance measurement was taken by the front sensor from the edge of the landing step above when the toe crosses the step during stair ascent and by the rear sensor from the edge of the landing step below when the heel crosses the step during stair descent.

An initial study found that IR-ToF sensors [23] can provide better accuracy than alternative technologies [24]. Furthermore, the sensor manufacturers (e.g., ST Microelectronics) only report the sensor performance under very specific and controlled conditions in the datasheet. Therefore, it is crucial to evaluate the system’s performance under working conditions while simulating real-life scenarios. For this specific study, the minimum foot clearance was generally less than 250 mm, so the measurement range was set to 0 mm to 250 mm.

The color of the target surface (dark grey carpet, light grey carpet, and wood) could potentially affect the accuracy associated with distance estimation, so different target surfaces were tested to cover the range of possible configurations occurring during stair walking. In static acquisitions, the target (carpet) was kept stationary in front of the ‘VL6180X distance sensor’ (fitted in the shoe). During dynamic acquisitions, the instrumented shoe was moved to the desired position.

The first distance experiment was performed with light gray carpets, dark gray carpets, and wood material. Next, the distance sensors were fitted to the shoes that were kept at 28 mm from the wood, dark gray, and light gray carpet. Similar results were found for all the materials tested. According to the VL6180X distance sensor’s datasheet, accuracy is ±3 mm; this is confirmed by this experiment as well.

Based on the results provided by the initial investigation into the influence of the target’s color, the light gray carpet was chosen for the subsequent experimental acquisitions. 

**Figure 8:**
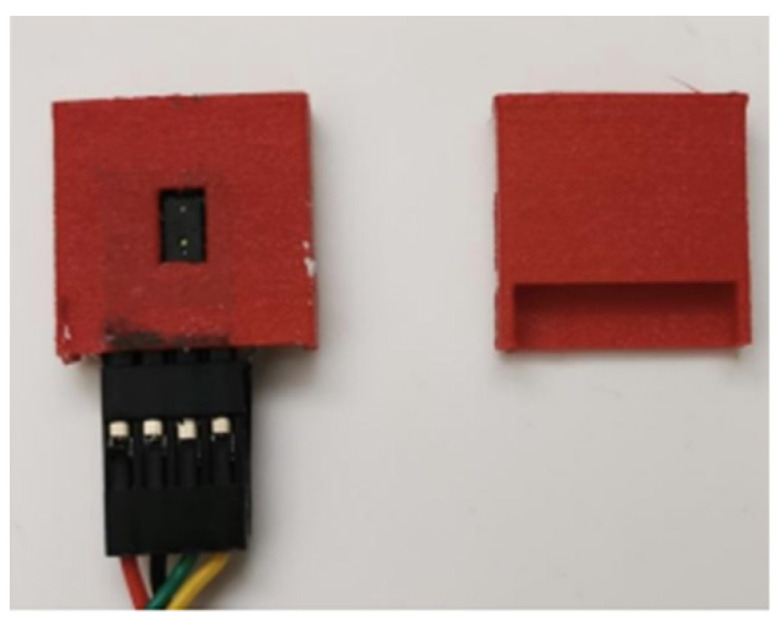
Distance sensor case.

In addition to FSRs not been affected by body weight as explained above the distance sensors would also not be affected by body weight. This is because they were fitted at the back of the shoe, inside a case that protected the distance sensors from potential demage. This case was designed using ‘solid works’ software and nylon material. Nylon filament is a reliable, durable, and versatile 3D printing material. Also, it is thin and flexible, with very high interlayer adhesion. The case was designed with a small rectangular opening because the distance sensor’s sensing module needs to see the object to measure the distance. Figure 8 shows the distance sensor case.

## 3. Experiment Setup

### 3.1. Participants 

Twenty-five older adults participated in this study (Age: 70.72 ± 4.0 years; body mass: 70.18 ± 10.0 kg; body height: 1.62 ± 0.06 m; female: 20; male: 5). All participants were recruited from the local communities of Wirral and Liverpool, UK and were living independently and able to climb stairs without help. The study was approved by the Liverpool John Moores University ethics committee in the UK (REF: 18/SPS/024). After the explained procedure, informed written consent was obtained from all participants.

### 3.2. Laboratory Measurements

A custom-made instrumented staircase was used to take measurements in the laboratory setting. This staircase was composed of seven steps in total, and force platforms were installed in the lower four steps from 1 to 4 to obtain kinetic data.

**Figure 9:**
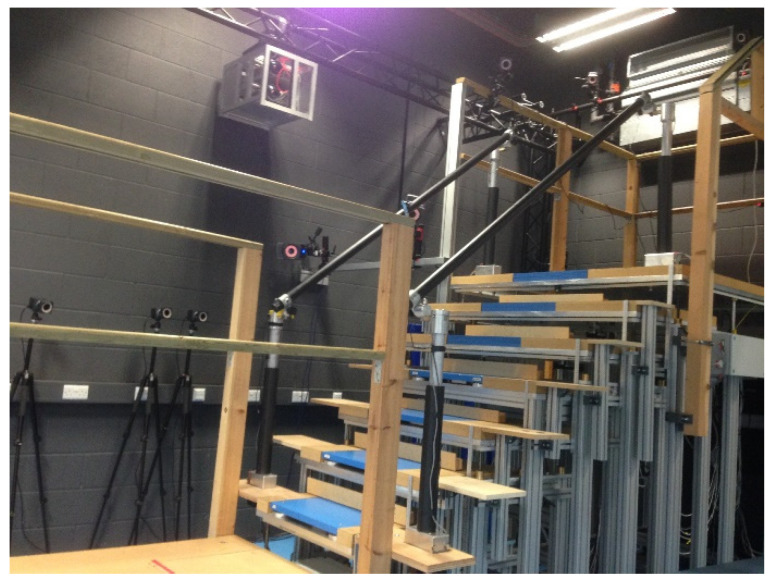
Laboratory custom-built instrumented seven-step staircase, first four steps fitted with a force plate, and twenty-four infrared cameras were used around the staircase for motion capture.

The custom-made staircase configuration was adjusted to match the stair dimensions of a typical private home, with the rise set at 19 cm and the flat run (going) at 23.5 cm. Handrails were placed on both sides of the staircase (Figure 9). The staircase was connected to the top, landing on one side and the walkway on the other side. Twenty-four infrared camera systems (Vicon, Oxford Metrics, UK) were used to obtain kinematic data. To measure force in the laboratory, ground reaction force platforms (9260AA, Kistler AG, Winterthur, Switzerland) were used.

### 3.3. Data Collection Procedure

The participants ascended and descended the instrumented staircase at their own preferred pace in a step-by-step manner, with handrails if needed. They were all dressed in tight-fitting Lycra shorts and shirts during stair negotiation and wore our instrumented shoes. A five-point safety harness was fitted to all participants, and this safety harness was attached to the overhead belay safety system to ensure safety. A trained member of the research team supported each participant’s overhead belay safety system by holding the safety rope system attached to the floor. Participants performed a few familiarization trials and then five trials, with the final three trials selected for further analysis.

### 3.4. Data Collection Using the Instrumented Shoes and the VICON System in the Custom-Made Laboratory Staircase

**Figure 10:**
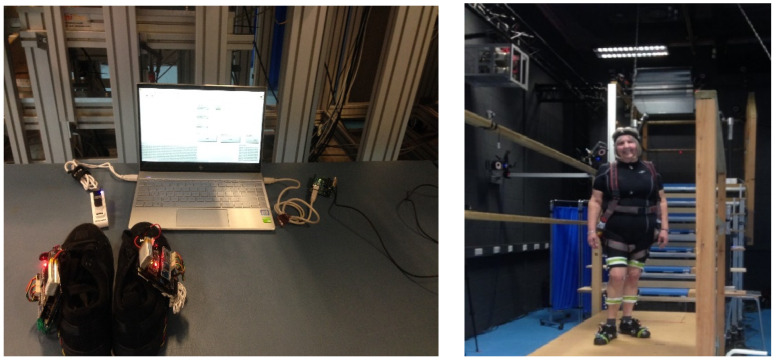
Lab data collection setup left side image was sensor shoe with computer connected with Bluetooth in one side of the computer, the other side of the computer connected with VICON system, right side image was participant with markers and sensor shoe.

Figure 10 shows the laboratory data collection setup and participants with markers attached and wearing instrumented shoes. In the laboratory, sensor data from the instrumented shoes were collected synchronously with the VICON system.

**Figure 11:**
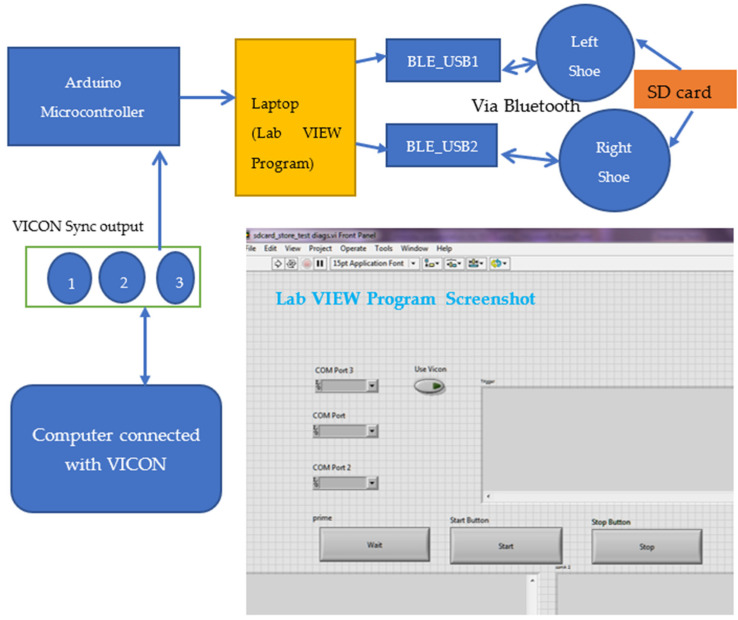
Instrumented shoes and VICON system synchronization. A computer was connected to the VICON system, and the VICON synchronization port 3 (port 1, 2 and 3) was connected to the Arduino microcontroller; a laptop connected to this Arduino microcontroller, contained a user interface to collect data from the instrumented shoes. Finally, two Bluetooth dongles were connected to the computer to signal the sensors in the shoes to start and stop data collection.

Figure 11 shows the synchronization process. There were three sync-out port in the Vicon system. To synchronize the VICON data with the sensors in the shoes, the VICON’s sync-out port three was connected to an Arduino microcontroller analog pin. Two Bluetooth (BLE-Bluetooth Low Energy) USPs (Universal Serial Port) connected with a laptop, and a LabVIEW user interface was programmed in the laptop computer to control the data collection from the sensors and store them into an ‘SD storage card’ with a ‘Unique file name’ for each data collection sequence. After completing all the trials with each participant, all the files were transferred to the computer from the SD card for further analysis.

### 3.5. Data Analysis-Instrumented Insole’s Foot Placement Calculation 

The foot placement ratio was calculated based on which sensor detected a force during contact with the step and then expressing the length of the insole covered by the loaded sensors as a percentage of the total insole length (Table 3 and Table 4). 

Different sizes of sensor insoles were created to fit different sizes of shoes, from 6 to 10. Each sensor insole had a different number of sensors. Shoe size 6 has got only six FSRs fitted, shoe size 7 has got seven sensors and so on. In the Table 3 and Table 4 shows shoe size and number of sensors for different shoe size. The number was given for each sensor from the toe as one to the heel as number nine (last number). 

**Figure 12:**
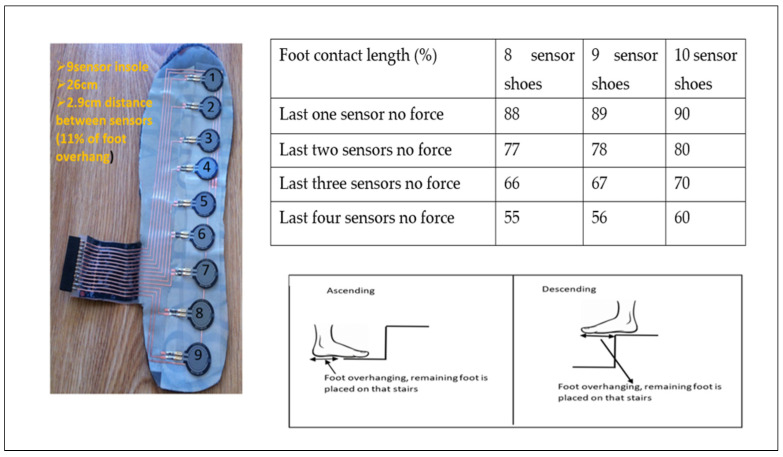
Foot placement ratio calculation.

The foot placement ratio was calculated as below: For example, Figure 12 shows an insole for a shoe size 9 with a total length of 26 cm and 9 force sensors. The distance between sensors in this insole was 2.9 cm, which was 11% of the whole insole length. If only the last sensor near the heel does not have any force (100% − 11% = 89%) during ascending, then 89% of the foot is placed on the stairs. Suppose the last two sensors do not have forces, which means (100% − 22% = 78%) 78% of the foot is placed on the stairs. If all the sensors have the forces, 100% of the foot is placed on the stairs. A similar process is followed when descending with the sensors at the front of the insole unloaded (foot overhang), depending on the percentage of the foot in contact with the step.

### 3.6. Data Analysis-Instrumented Shoes Foot Clearance Calculation 

After data collection, foot clearance was calculated using a method explained in [22]. 

**Figure 13:**
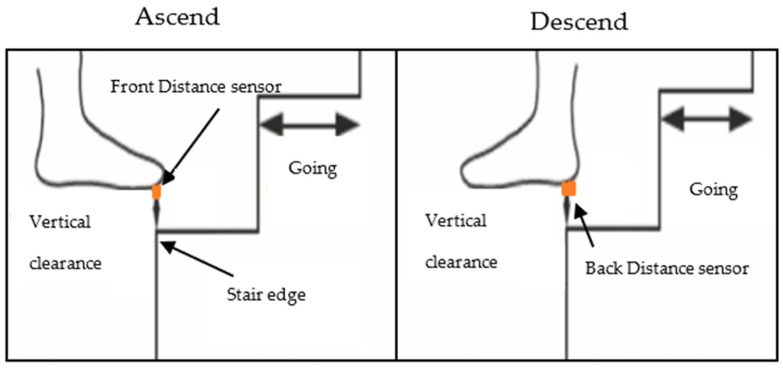
Instrumented shoe’s foot clearance calculation.

Briefly, for descending, during the swing phase, foot clearance was calculated when the sensor shoe’s back distance sensor of the leading limb (Figure 13) passed the vertical position of the step edge and for ascending, during the swing phase, when the sensor shoe’s front distance sensor of the leading limb passed the second step edge before placing the foot on the stairs. 

**Figure 14:**
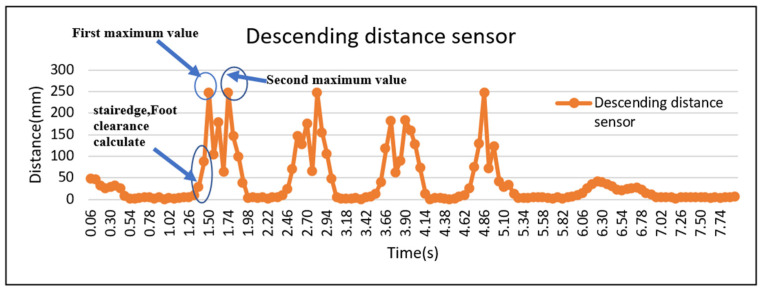
Graphical representation of minimum foot clearance calculation while descending.

Figure 14 shows the foot clearance calculation graph for descending. While descending, the foot must cross two stair edges. While crossing the first step edge, the minimum foot clearance was calculated. After crossing the first edge, the sensor reaches the first maximum and goes down, and after crossing the second stair edge, the sensor reaches the second maximum value and goes down to land. MATLAB R2016a was used to calculate the minimum foot clearance using the above information.

### 3.7. Data Analysis-VICON Systems Foot Placement and Foot Clearance Calculation

**Figure 15:**
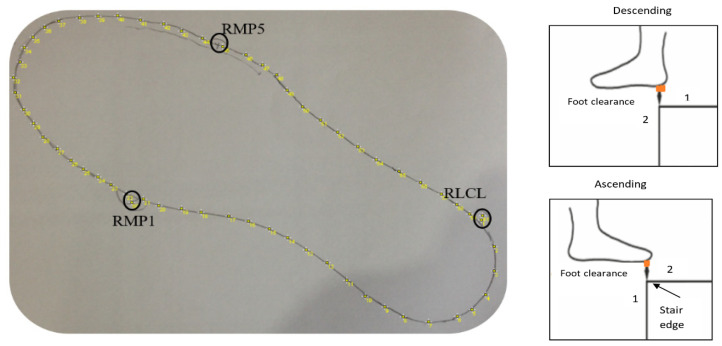
VICON foot clearance calculation of the right foot. 1 and 2 are the positions of the virtual shoe.

VICON foot placement and foot clearance were calculated using the data analysis method described before [6,8]. So, this paper provides only a summary of VICON’s data analysis method. Foot clearance and foot placement ratio data were obtained using a 24-infrared camera system (VICON) and force plates. The participant’s sensor shoes were digitalized manually. The participant’s sensor shoe’s two-dimensional outline was obtained by taking a picture of the participants’ sensor shoe outline drawn on an A4 paper (Figure 15) and importing it using ImageJ V1.43 (National Institutes of Health, Bethesda, MD, USA). The coordinates of up to 600 virtual markers representing the individual shoe sole outline were then calculated in MATLAB. The positions of three markers fixed on the shoe (first metatarsophalangeal joint (RMP1), fifth metatarsophalangeal joint (RMP5), and calcaneus lateral (RLCL)) were digitized in the two-dimensional drawing and used for static measurement. These static measurements included the above three marker positions in a 3D (three-dimensional) space, which helps determine the positions of the shoe’s virtual outline relative to the markers. The virtual outline of the shoe was then projected in movement trials, again relative to the three markers.

The foot clearance (Figure 15) was obtained during the swing phase when the virtual shoe outline of the leading limb passed the vertical position (1) of the step edge up until the outline passed the horizontal position of the step edge (2). The minimal clearance of the virtual shoe was determined within this time frame. The minimal foot clearance was determined for steps 1–7 in all three trials. The mean value across the three trials was considered for further analysis.

**Figure 16:**
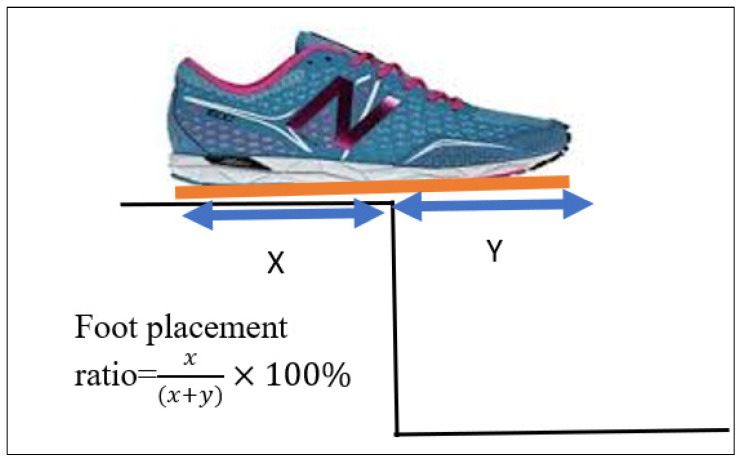
VICON foot placement ratio calculation.

We calculated the foot placement ratio using the foot touchdown over the ‘force plate’, placed on steps 1–4. Distance X was measured, the distance between the step edge and the posterior foot end of the virtual shoe line, and distance Y, the distance between the step edge and the most anterior foot end of the virtual shoe outline (Figure 16).

The foot placement ratio was calculated using the formula $\frac{x}{\left(x+y\right)}\times 100\%$. The mean value across three trials was calculated and used for further analysis.

## 4. Statistics 

To assess the agreement between smart shoe lab-based outcomes, we performed three different tests.

Test 1: Quantification of the correlation coefficient (r) between smart shoes and lab-based outcomes; Test 2: Quantification of the coefficient of determination (r^2^) and regressions between the two techniques to find variance and the best-fit line; Test 3: Bland–Altman plots to quantify the agreement between smart shoe and lab-based parameters.

The correlation is used to check the relationship between two variables, and it shows how strongly they are related. The most common correlation techniques are Pearson correlation and product-moment correlation, and this correlation result is called the correlation coefficient(r). It is calculated as the covariance ratio between the variables and the product of their standard deviations. The correlation coefficient(r) ranges from +1 to −1. This value helps to get an idea of the relationship strength; the correlation coefficient(r) value closer to +1 shows a strong positive linear relationship, while close to −1 shows a strong negative linear relationship. R square is the coefficient of determination and indicates the percentage of variation explained by the regression line out of the total variation.

The correlation coefficient describes the relationship between two variables (sensor and VICON), but it does not describe their agreement. A high correlation does not mean a good agreement between the two variables. So, the Bland–Altman plot was used to measure the agreement between two variables by constructing limits of agreement. These limits of agreement were calculated using the mean and standard deviations of the difference between sensor and VICON fall risk measurements [25].

Foot placement ratios’ coefficient of determination was calculated for all four force plates in the three trials (N = 75, 3 trials × 25 participants). The mean value of the three trials’ foot placement ratio was used to calculate the overall coefficient of determination and regression line (25 participants × 4 steps = 100 data points), correlation coefficient, and Bland-Alman plot.

Each participant’s three trials that were included in the analysis were used to calculate each step’s correlation coefficients for foot clearance between the different techniques (25 participants × 3 trials = 75 N data points for each step). The mean value of three trials’ foot clearance was used to calculate the overall coefficient of determination, regression line, correlation coefficient (25 participants × 7 steps = 175 data points), and Bland–Altman plots.

The correlation coefficients describe the relationship between two variables (sensor and VICON parameters), but they do not describe their agreement [26]. A high correlation does not mean a good agreement between two variables. So, the Bland–Altman plot was used to measure the agreement between the two variables by constructing limits of agreement [26]. These limits of agreement were calculated using the mean and standard deviations of the difference between the shoe sensor and the VICON foot placement ratio and foot clearance measurements.

## 5. Results and Discussion

In the present study, we developed and validated smart shoes instrumented with sensors to estimate foot contract length and foot clearance during stair negation in real-life staircases. The validation procedure involved three tests.

*Test 1: Descending correlation coefficients (r) to quantify the relationship between sensor and VICON outputs.*

Pearson correlation was used to quantify the relationships for foot placement and clearance. The results are presented in Table 5 and Table 6.

The Pearson correlation for foot placement ratio (FO) between the shoe sensor and VICON was = 0.878 (*p* < 0.001).

The Pearson correlation for foot clearance (FC) between the shoe sensor and VICON was r = 0.967 (*p* < 0.001). These results show excellent agreement between shoe sensors and VICON outputs.

*Test 2: Coefficient of determination (r^2^) and regressions to find the variance and best-fit lines.*

**Figure 17:**
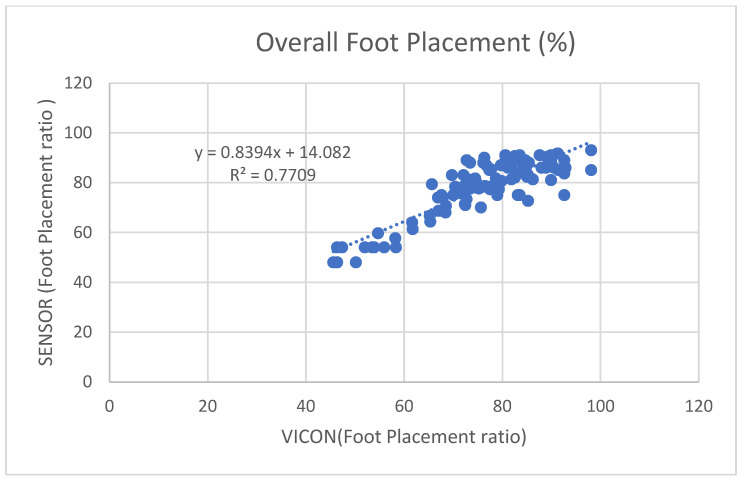
Four steps combined foot placement ratio results between sensors and VICON.

**Figure 18:**
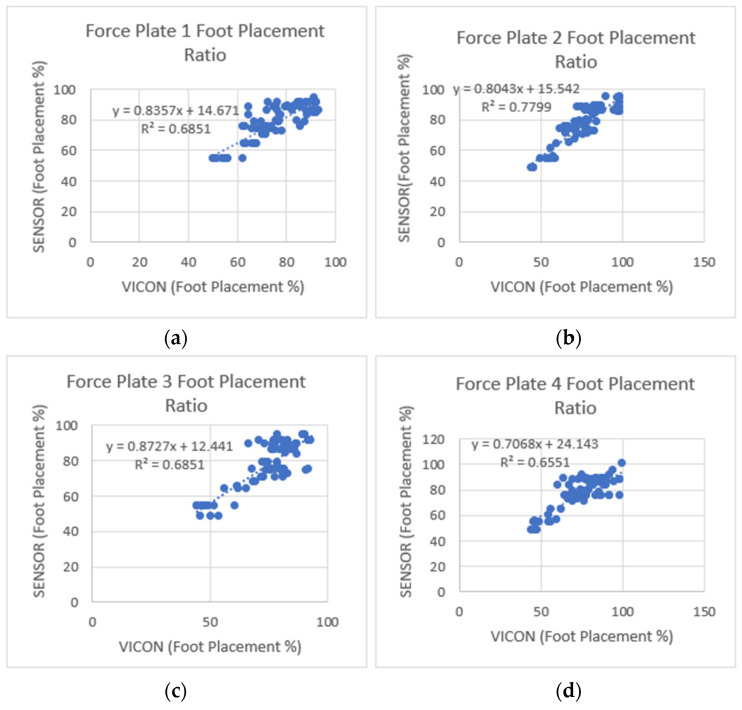
Foot placement ratio’s coefficient of determination ($r^{2}$) and regression results between the sensor and VICON (laboratory system). (**a**) Step one-foot placement ratio, (**b**) step two-foot placement ratio, (**c**) step three-foot placement ratio, and (**d**) step four-foot placement ratio. Among the four steps, step two showed a higher positive linear coefficient of determination between the sensors and the VICON system.

Figure 17 shows the overall coefficient of determination $r^{2}=0.77$, so 77% of the variation in the sensor’s foot placement is predicted by the statistical model, and 77% of the variance is shared between the sensor’s and VICON’s foot placement ratio. Figure 18 shows that Force Plates 1, 3, and 4 had a moderate coefficient of determination ($r^{2}=$0.6851, 0.6851, and 0.6551), and Force Plate 2 (FP2) had a high positive coefficient of determination (0.7799). Linear regression was calculated to find the best line that predicts the sensor shoe placement ratio from the VICON placement ratio. The calculated linear regression results are in Figure 17 and Figure 18.

**Figure 19:**
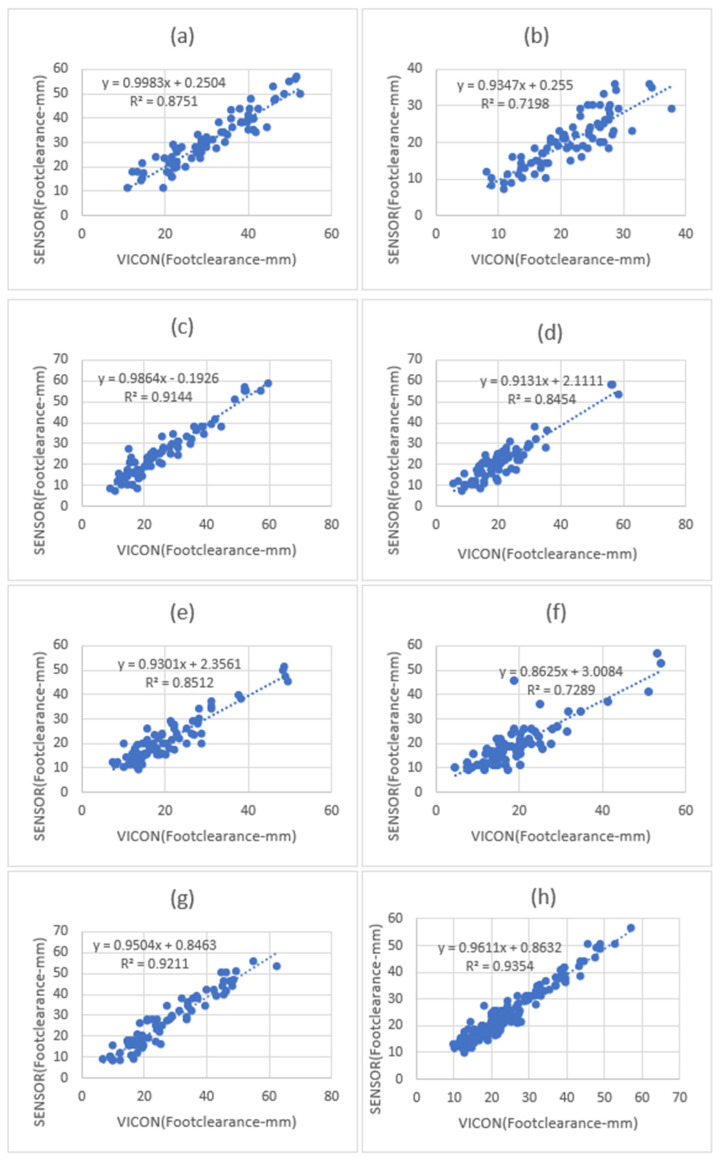
Foot clearance coefficient of determination (r^2^) and regression line results between sensors and VICON (laboratory system). (**a**) Step one-foot clearance, (**b**) step two-foot clearance correlation, (**c**) step three-foot clearance, (**d**) step four-foot clearance, (**e**) step five clearance correlation, (**f**) step six-foot clearance, and (**g**) step seven-foot clearance. (**h**) All seven steps combined foot clearance. Step one, step seven, and all seven combined foot clearances showed a high positive linear coefficient of determination between the sensors and the VICON system.

Figure 19 shows the overall coefficient of determination was $r^{2}=$ 0.96, so 96% of the variation in the sensor’s foot clearance is predicted by the statistical model, and 96% of the variance is shared between the sensor’s and VICON’s foot clearance. Step 3 and Step 7 had a high positive coefficient of determination (0.9144, 0.9211), while steps 2 and 6 had slightly fewer positive coefficients of determination (0.7198, 0.7289). Step 1, Step 4, and Step 5 (0.8751, 0.8454, and 0.8512) had a better coefficient of determination than Step 2 and Step 6 and less than Step 3 and Step 7. Linear regression was calculated to find the best line that predicts sensor foot clearance from the VICON foot clearance. The calculated linear regression line results are shown in Figure 19.

*Test 3: Bland–Altman plots to check the agreement between the sensor and VICON parameters.*

**Figure 20:**
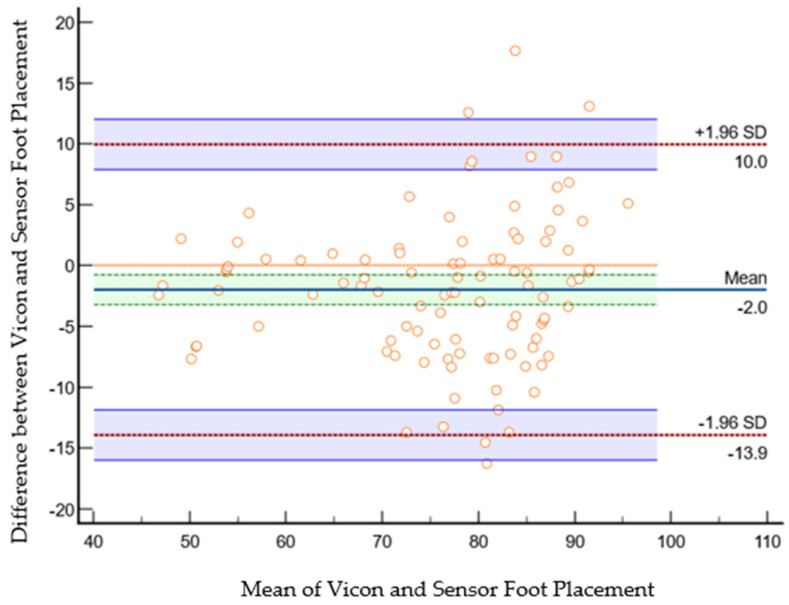
Bland–Altman plot of foot placement ratio agreement between the sensor method and the VICON method in the laboratory. The X-axis represents the average of the sensor and VICON foot placement ratio; the Y-axis represents the difference between the sensor and VICON foot placement ratios. The indigo horizontal line denotes the mean difference between sensor and VICON methods, which was −2%, while the upper red line represents the upper limits of agreement (mean difference + 1.96 × SD of difference), which was 10%, and the lower red line represents the lower limits of agreement (mean difference − 1.96 × SD of difference), which was −13.9%. The purple lines show the confidence limits for the upper and lower limits of agreement. The small orange circle are data points; when shown outside of the confidence limits, they are considered outliers; they are four outliers in total (4 out of 100 datapoints). The orange horizontal line shows the line of equality; it is possible to determine if bias is significant or not. This equality line was not within the confidence interval of the mean difference, so the bias is significant (−2%).

The scatter plot in Figure 20 shows Bland–Altman’s agreement results for the sensor and VICON foot placement ratio. Plotting the difference against the mean value allows us to quantify the relationship between measurement error and actual value [26]. The average of the differences was −2% (bias), which means that, on average, the second method (sensor shoe) underestimates by the 2%-foot placement ratio compared to the first method (VICON). Repeatability is the degree to which the same method produces the same results on repeated measurements. The standard deviation (SD) of all the individual differences was calculated to measure repeatability, 6.08%. Approximately 4% (4 of 100 data points) of the data points were outliers and exceeded the upper and lower limits of agreement. The Shapiro–Wilk method was used to check the normality of the data point in differences (VICON foot placement ratio sensor foot placement ratio), and the results (*p* = 0.266) showed that differences were normally distributed. Limits of the agreement were calculated; 95% of the limits of agreement were between 10% (upper limit) and −13.91% (lower limit). Precision is the degree (confidence limit) to which values cluster around the mean distribution of values, which was the 95% confidence limit for the upper (7.8% to 12%) and the 95% confidence limit for the lower limits of agreement (−15.97% to −11%). Therefore, the foot placement ratio results measured by the VICON system were −13.91% less or 10% more than sensor-based measurements. This confidence limit was small enough to ensure that the new method (shoe sensor) could calculate the foot placement ratio instead of the VICON method.

**Figure 21:**
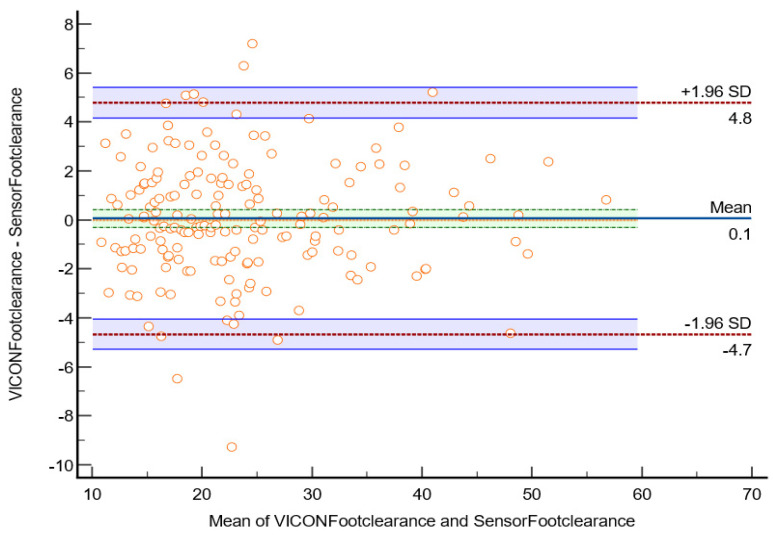
Bland–Altman plot of foot clearance between the sensor method and the VICON method in the laboratory. The X-axis represents the average of the sensor and VICON foot clearance ratio; the Y-axis represents the difference between the sensor and VICON foot clearance. The indigo horizontal line denotes the mean difference between sensor and VICON methods, which was 0.1 mm, while the upper top horizontal red line represents the upper limits of agreement (mean difference + 1.96 × SD of difference), which was 4.8 mm, the lower red line represents the lower limits of agreement (mean difference − 1.96 × SD of difference), which was −4.7 mm. The purple lines show the confidence limits for the upper and lower limits of agreement. The small orange circles are data points, when shown outside of the confidence limits, they are considered outliers; there are four outliers in total (4 out of 175 datapoints). The orange horizontal line shows the line of equality; it is possible to determine if bias is significant or not. This equality line was not within the confidence interval of the mean difference, so the bias is significant (0.1 mm).

The scatter plot in Figure 21 shows Bland–Altman’s agreement results for sensor and VICON foot clearance measurements to quantify the relationship between measurement error and actual value [26]. The average of the differences was 0.05 mm (bias), which indicates that, on average, the second method (sensor shoe) overestimates by 0.0566 mm foot clearance compared to the first method (VICON). Repeatability is the degree to which the same method produces the same results on repeated measurements. The standard deviation (SD) of all the individual differences was calculated to measure repeatability, 2.41 mm. Approximately 5% of data points (10 of 175 data points) were outliers and exceeded the upper and lower limits of agreement. The Shapiro–Wilk method was used to check the normality of the data point in differences (VICON foot clearance data-sensor foot clearance data). The results (*p* = 0.210) showed that differences were normally distributed. The limit of agreement was calculated; 95% of the limits of agreement were between the upper limit (4.8 mm) and lower limit (−4.7 mm). Precision is the degree (confidence limit) to which values cluster around the mean distribution of values, which was the 95% confidence limit of the upper (4.1 mm to 5.4 mm) and lower (−5.2 mm to −4.0 mm) limits. The parameters measured by the VICON system may be 4.8 mm above or 4.7 mm below the sensor measurement of foot clearance. This confidence limit was small enough to provide confidence that the new method (instrumented shoe sensors) could be used to calculate foot clearance instead of the VICON system.

*Test 1: Ascending correlation coefficients (r) to quantify the relationship between sensor and VICON outputs.*

Pearson correlation was used to quantify the relationships for foot placement and clearance. The results are presented in Table 7 and Table 8.

The Pearson correlation for foot placement ratio between the sensor shoe and VICON was =0.838 (*p* < 0.001).

The Pearson correlation for foot clearance between the shoe sensor and VICON was =0.843 (*p* < 0.001). These results show excellent agreement between shoe sensors and VICON outputs.

*Test 2: Coefficient of determination (r^2^) and regressions to find the variance and best-fit lines.*

**Figure 22:**
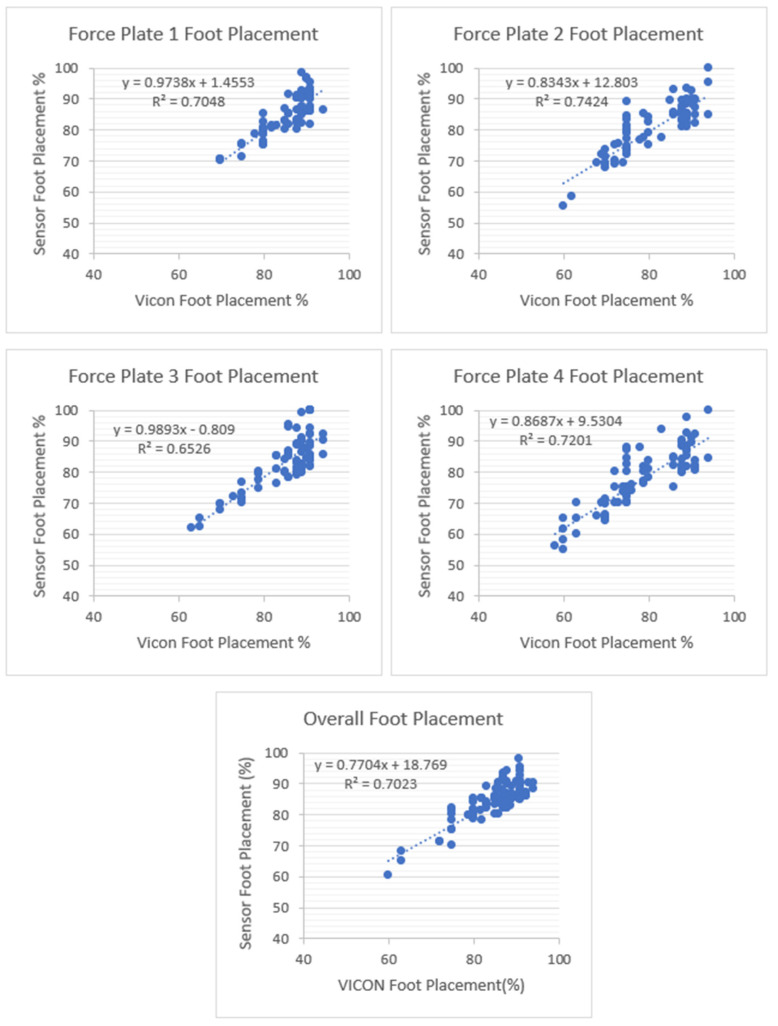
Foot placement ratio’s coefficient of determination ($r^{2}$) and regression results between the sensor and VICON (laboratory system). There are four force plates (FPs) in the laboratory. All four force plates’ foot placement ratios were calculated to measure the coefficient of determination between sensors and VICON. Among the four steps, step two showed a higher positive linear coefficient of determination between the sensors and the VICON system.

Figure 22 shows the overall coefficient of determination $r^{2}=0.70$, so 70% of the variation in the sensor’s foot placement is predicted by the statistical model, and 70% of the variance is shared between the sensor’s and Vicon’s foot placement ratio. Force Plate 3 had a moderate coefficient of determination ($r^{2}=$0.6526) and FP 1, 2, and 4 had high positive coefficients of determination (0.7048, 0.7424, and 0.7201). Linear regression was calculated to find the best line that predicts the sensor shoe foot placement ratio from the VICON foot placement ratio. The calculated linear regression results are in Figure 22.

**Figure 23:**
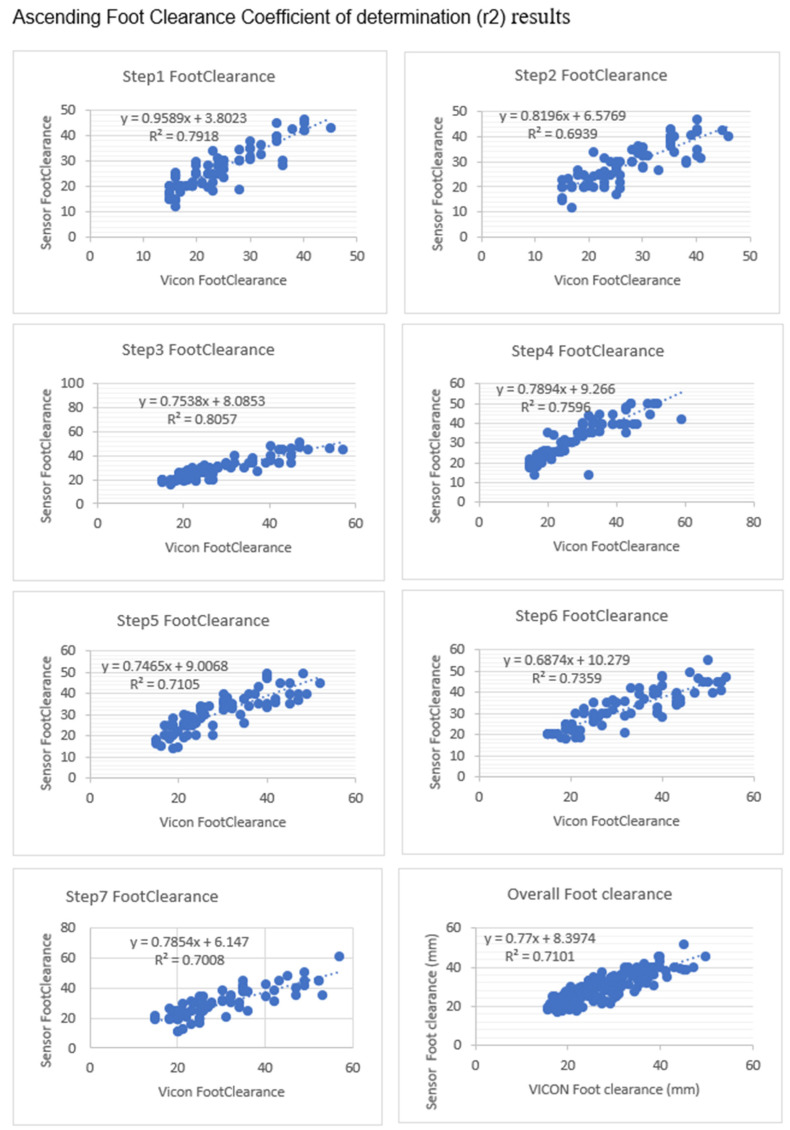
Foot clearance coefficient of determination (r^2^) and regression line results between sensors and VICON (laboratory system). There are seven steps in the laboratory; each step’s foot clearance was calculated along with the overall foot clearance. Step1’s and Step3’s foot clearance showed a high positive linear coefficient of determination between the sensors and the VICON system.

Figure 23 shows the overall coefficient of determination was $r^{2}=$0.71, so 71% of variation in the sensor’s foot clearance is predicted by the statistical model, and 71% of the variance is shared between the sensor’s and VICON’s foot clearance. Step 1 and Step 3 had a high positive coefficient of determination (0.7918 and 0.8057), while Step 2 and Step 5 had slightly fewer positive coefficients of determination (0.6939 and 0.7105). Step 4 and Step6 (0.7596 and 0.7539) had a better coefficient of determination than Step 2 and Step5 and less than Step 1 and Step 3. Linear regression was calculated to find the best line that predicts sensor foot clearance from the VICON foot clearance. The calculated linear regression line results are in Figure 23.

*Test 3: Ascending Bland–Altman plots to check the agreement between the sensor and VICON parameters.*

**Figure 24:**
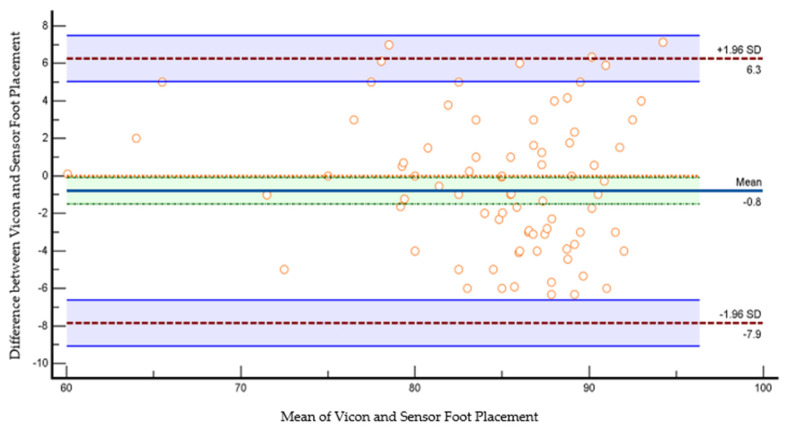
Bland–Altman plot of foot placement ratio agreement between the sensor method and the VICON method in the laboratory. The X-axis represents the average of the sensor and VICON foot placement ratio; the Y-axis represents the difference between the sensor and VICON foot placement ratios. The indigo horizontal line denotes the mean difference between sensor and VICON methods, which was −0.8%, while the upper red line represents the upper limits of agreement (mean difference + 1.96 × SD of difference), which was 6.3%, and the lower red line represents the lower limits of agreement (mean difference − 1.96 × SD of difference), which was −7.9%. The purple lines show the confidence limits for the upper and lower limits of agreement. The small orange circles are data points. The orange horizontal line shows the line of equality; it is possible to determine if bias is significant or not. This equality line was not within the confidence interval of the mean difference, so the bias is significant (−0.8%).

The scatter plot in Figure 24 shows Bland–Altman’s agreement results for the sensor and VICON foot placement ratio. Plotting the difference against the mean value allows us to quantify the relationship between measurement error and actual value [26]. The average of the differences was −0.8% (bias), which means that, on average, the second method (sensor shoe) underestimates by 0.8% the foot placement ratio compared to the first method (VICON). Repeatability is the degree to which the same method produces the same results on repeated measurements. Limits of the agreement were calculated; 95% of the limits of agreement were between 6.3% (upper limit) and −7.9% (lower limit). Precision is the degree (confidence limit) to which values cluster around the mean distribution of values, which was the 95% confidence limit for upper (5–7.4%) and 95% confidence limit for lower limits of agreement (−9% to −6%). Therefore, the foot placement ratio results measured by the VICON system are −7.9% less or 6.3% more than the sensor-based measurements. This confidence limit was small enough to ensure that the new method (shoe sensor) could calculate the foot placement ratio instead of the VICON method.

**Figure 25:**
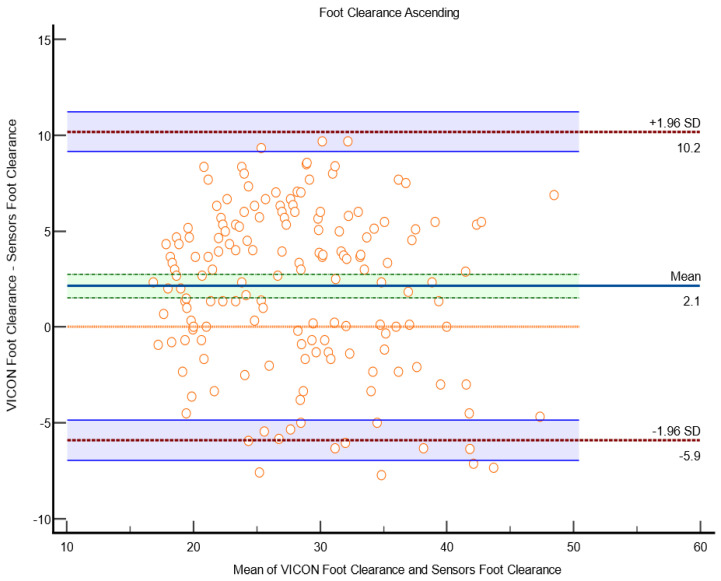
Bland–Altman plot of foot clearance between the sensor method and the VICON method in the laboratory. The X-axis represents the average of the sensor and Vicon foot clearance ratio; the Y-axis represents the difference between the sensor and VICON foot clearance. The indigo horizontal line denotes the mean difference between the sensor and VICON methods, which was 2.1 mm, while the upper top horizontal red line represents the upper limits of agreement (mean difference + 1.96 × SD of difference), which was 10.2 mm, and the lower red line represents the lower limits of agreement (mean difference − 1.96 × SD of difference), which was −5.9 mm. The purple lines show the confidence limits for the upper and lower limits of agreement. The small orange circle are data points, when shown out of the confidence limits; they are considered outliers; four outliers were in total (4 out of 175 datapoints). The orange horizontal line shows the line of equality; it is possible to determine if bias is significant or not. This equality line was not within the confidence interval of the mean difference, so the bias is significant (2.1 mm).

The scatter plot in Figure 25 shows Bland–Altman’s agreement results for sensor and VICON foot clearance measurements to quantify the relationship between measurement error and actual value [26]. The average of the differences was 2.1 mm (bias), which indicates that, on average, the second method (sensor shoe) overestimates by 2.1 mm foot clearance compared to the first method (VICON). Repeatability is the degree to which the same method produces the same results on repeated measurements. The standard deviation (SD) of all the individual differences was calculated to measure repeatability, 9 mm. Approximately 2% of data points (4 of 175 data points) were outliers and exceeded the upper and lower limits of agreement. The limit of agreement was calculated; 95% of the limits of agreement were between the upper limit (10 mm) and lower limit (−5 mm). Precision is the degree (confidence limit) to which values cluster around the mean distribution of values, which was the 95% confidence limit of upper (9.1 mm to 11 mm) and lower (−6.9 mm to −4.8 mm). The parameters measured by the VICON system may be 10 mm above or −5.9 mm below the sensor measurement of foot clearance. This confidence limit was small enough to provide confidence that the new method (instrumented shoe sensors) could be used to calculate foot clearance instead of the VICON system.

### Difference between Ascending and Descending Correlation and Agreement Results

Table 9 shows the difference between ascending and descending correlation and agreement results. The ascending foot placement ratio had better agreement than the descending one. A similar correlation and coefficient of determination were found in both ascending and descending foot placement ratios. On the other hand, descending foot clearance had a better correlation, coefficient of determination, and agreement than ascending foot clearance.

In conclusion, an instrumented shoe system prototype was developed to calculate foot placement and foot clearance during stair negotiation on real-life staircases. The sensor output showed good agreement against the golden standard biomechanical measurements taken in an experimental staircase in a gait lab, thus offering promise for advancing the current prototype into a measurement device for estimating fall risk in real-life staircases. Two low-cost sensors were used to create the instrumented shoe: a vl6180x distance sensor and an FSR; each sensor shoe cost 200 EUR to develop, so the total cost for a pair of shoes was 400 EUR.

The current instrumented setup could only measure vertical foot clearance, not horizontal foot clearance. However, horizontal clearance is also necessary while descending stairs to reduce the risk of slips. Therefore, this project’s printed circuit board can accommodate a few more distance sensors. With this ability, we can fit the distance sensor at the shoe’s back (heel counter) to find horizontal foot clearance while descending stairs. We tried to include the distance sensor at the back (heel counter) of the shoe, but the data collection frequency went down, and it was hard to fit it appropriately in place. In the future, we will seek alternative solutions to calculate horizontal clearance.

## Figures and Tables

**Table 1:** Relevant parameters of the interlink FSR-402.

Parameter	Value
Force sensitivity range	<100 g to >10 kg depending on mechanics
Pressure sensitivity range	<0.1 kg/cm^2^ to >10 kg/cm^2^
Part-to-part repeatability	±15% to 25% of established nominal resistance
Single part repeatability	±2% to 5% of established nominal resistance
Current consumption	1 mA/cm^2^ of applied force
Resolution	0.5% full scale
Temperature Range	−30 °C to +70 °C
Sensitivity to noise/vibration	Not significantly affected
Devices rise time	1.2 ms
Lifetime	>10 million actuations
Hysteresis	+10% average

**Table 2:** Distance sensor specification.

Feature	Details
Size	0.5″ × 0.7″ × 0.085″ (13 mm × 18 mm × 2 mm)
Mass	0.5 g (0.02 oz)
Operating voltage	2.7 V to 5.5 V
Output format (I²C)	8-bit distance reading
Distance measuring range	0 mm to 250 mm
Resolution	1 mm
Temperature-dependent drift	15 mm
Accuracy	±3 mm
Noise	2.0 mm maximum
Frequency	30 Hz
Laser-Infrared Emitter	850 nm

**Table 3:** Foot placement calculation when ascending.

Foot Placement % Ascend										
Shoe Size	10th FSR	9th FSR	8th FSR	7th FSR	6th FSR	5th FSR	4th FSR	3rd FSR	2nd FSR	1st FSR
6	No FSR	No FSR	No FSR	No FSR	100	83	66	49	32	15
7	No FSR	No FSR	No FSR	100	86	72	57	43	29	15
8	No FSR	No FSR	100	88	75	63	50	38	25	13
9	No FSR	100	89	78	67	56	45	34	23	12
10	100	90	80	70	60	50	40	30	20	10

**Table 4:** Foot placement calculation when descending.

Foot Placement % Descend										
Shoe Size	1st FSR	2nd FSR	3rd FSR	4th FSR	5th FSR	6th FSR	7th FSR	8th FSR	9th FSR	10th FSR
6	100	83	66	49	32	15	No FSR	No FSR	No FSR	No FSR
7	100	86	72	57	43	29	15	No FSR	No FSR	No FSR
8	100	88	75	63	50	38	25	13	No FSR	No FSR
9	100	89	78	67	56	45	34	23	12	No FSR
10	100	90	80	70	60	50	40	30	20	10

**Table 5:** Foot placement ratio Pearson correlation results sensor and VICON.

Pearson Correlations			
		Foot Placement VICON	Foot Placement SENSOR
Foot placement for VICON	Pearson Correlation	1	0.878 **
	Sig. (2-tailed)		0.000
	N	100	100
Foot placement for sensor	Pearson Correlation	0.878 **	1
	Sig. (2-tailed)	0.000	
	N	100	100

** Correlation is significant at the 0.01 level (2-tailed).

**Table 6:** Foot clearance Pearson correlation result between sensors and VICON.

Pearson Correlations(r)			
		Foot Clearance VICON	Foot Clearance SENSOR
Foot Clearance VICON	Pearson Correlation	1	0.967 **
	Sig. (2-tailed)		0.000
	N	175	175
Foot Clearance SENSOR	Pearson Correlation	0.967 **	1
	Sig. (2-tailed)	0.000	
	N	175	175

** Correlation is significant at the 0.01 level (2-tailed).

**Table 7:** Pearson correlation results for foot placement when ascending.

Pearson Correlations			
		Sensor Foot Contact Length	VICON Foot Contact Length
Foot contact length for Sensor	Pearson Correlation	1	0.838 **
	Sig. (2-tailed)		<0.01
	N	100	100
Foot placement for Vicon	Pearson Correlation	0.838 **	1
	Sig. (2-tailed)	<0.01	
	N	100	100

** Correlation is significant at the 0.01 level (2-tailed).

**Table 8:** Pearson correlation results for foot clearance when ascending.

Pearson Correlations(r)			
		Foot Clearance VICON	Foot Clearance Sensor
Foot Clearance VICON	Pearson Correlation	1	0.843 **
	Sig. (2-tailed)		<0.01
	N	175	175
Foot Clearance SENSOR	Pearson Correlation	0.843 **	1
	Sig. (2-tailed)	<0.01	
	N	175	175

** Correlation is significant at the 0.01 level (2-tailed).

**Table 9:** Difference between ascending and descending correlation and agreement results.

Negotiation	Descending	Ascending
Foot placement ratio	R = 0.878	R = 0.838
	Overall r^2^ = 0.77	Overall r^2^ = 0.70
	Forceplate1 r^2^ = 0.68	Forceplate1 r^2^ = 0.70
	Forceplate2 r^2^ = 0.77	Forceplate2 r^2^ = 0.74
	Forceplate3 r^2^ = 0.68	Forceplate3 r^2^ = 0.65
	Forceplate4 r^2^ = 0.65	Forceplate4 r^2^ = 0.72
	Bias = −2%	Bias = −0.8%
	Upper limit = 10%	Upper limit = 6.3%
	Lower limit = −13.9%	Lower limit = −7.9%
Foot clearance	R = 0.967	R = 0.843
	Step 1 r^2^ = 0.87	Step 1 r^2^ = 0.79
	Step 2 r^2^ = 0.71	Step 2 r^2^ = 0.69
	Step 3 r^2^ = 0.91	Step 3 r^2^ = 0.80
	Step 4 r^2^ = 0.84	Step 4 r^2^ = 0.75
	Step 5 r^2^ = 0.85	Step 5 r^2^ = 0.71
	Step 6 r^2^ = 0.72	Step 6 r^2^ = 0.73
	Step 7 r^2^ = 0.92	Step 7 r^2^ = 0.70
	Overall r^2^ = 0.96	Overall r^2^ = 0.71
	Bias = 0.1 mm	Bias = 2.1 mm
	Upper limit = 4.8 mm	Upper limit = 10.2 mm
	Lower limit = −4.7 mm	Lower limit = −5.9 mm

## Data Availability

Data are contained within the article and supplementary materials.

## Supplementary Materials

The following supporting information can be downloaded at: https://www.mdpi.com/article/10.3390/s23249638/s1. Figure S1. FSR Calibration with Tinius Olsen material testing machine; Figure S2. Calibration Curve for higher force (Leftside) to measure 30 N to 100 N and lower force (rightside) to measures 1 N to 30 N. After designing the circuit with Op-Amp, the FSR was calibrated with the materials testing machine; Figure S3. Distance sensor calibration curve, sensor angle set for zero degrees to 60 degrees by increasing it in 10° (0°, 10°, 20°, 30°, 40°, 50°, 60°), at the same distance was measured for 10 mm, 15 mm, 20 mm, 25 mm, 30 mm, 35 mm and 40 mm. Linear correlation R^2^ = 0.9226; Table S1. Distance sensor offset and accuracy testing results.
