# Supplementary material for: A Novel Smart Shoe Instrumented with Sensors for Quantifying Foot Placement and Clearance during Stair Negotiation
Source: Sensors (Basel). 2023 Dec 5;23(24):9638. doi: 10.3390/s23249638 (PMC10747814; doi:10.3390/s23249638)
Supplement: Supplementary file 1 [file sensors-23-09638-s001.zip › sensors-2571344-supplementary.pdf]

Supplementary Materials

1. FSR Calibration

The zero offset was not required. Hence the FSRs are loaded under the foot inside a shoe with a snugly tied lace. Therefore, the load on FSRs when the foot is in the air is not expected to be 0N. So long as the sensitivity is determined at small values close to 0N, zero offsets are not required. These FSR outputs are non-linear, and their single part repeatability is from ±2% to ±5% of the nominal resistance.

The FSR output was calibrated to the units of force using the applied mass. Each FSR was tested in the material testing machine (‘Tinius Olsen material testing machine’) that was used to apply successive forces from 0N to 100 N, continually recording the actual applied force and the corresponding outputs of the FSRs simultaneously (Figure S1).

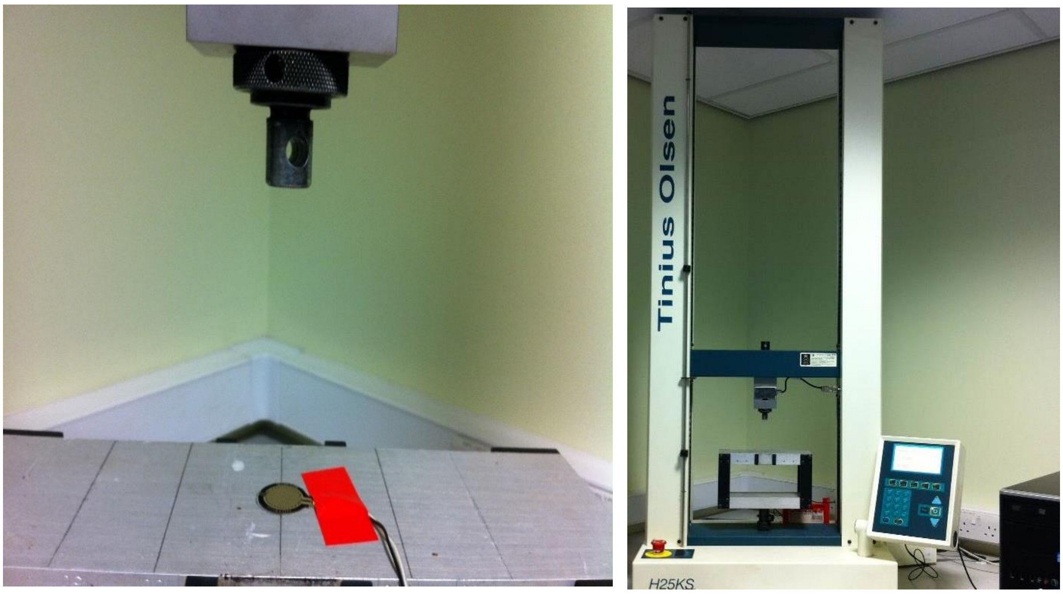

Figure S1. FSR Calibration with Tinius Olsen material testing machine.

Figure S2 shows the calibration results, FSR higher and lower forces voltage vs material testing machine forces, with a second x-axis showing the equivalent applied force unit from the testing machine.

To capture the non-linear relationship between the FSR higher force output and applied force, fourth-order Polynomial curve fitting y = 3E-05x4 - 0.0023x3 + 0.0706x2 - 0.2793x + 32.803 was used for calibration and the R squared value for this curve fitting was 0.9984 showing the excellent reliability of the sensors (Figure S2 left side).

Figure S2 (right side) also shows the non-linear relationship between the FSR lower force output and applied force using a third-order Polynomial curve fitting y = 6E-06x3 - 0.0019x2 + 0.1923x -3.6929 that was used for calibration. The R squared value for this curve fitting was R^2 = 0.9766 showing the excellent reliability of the sensors.

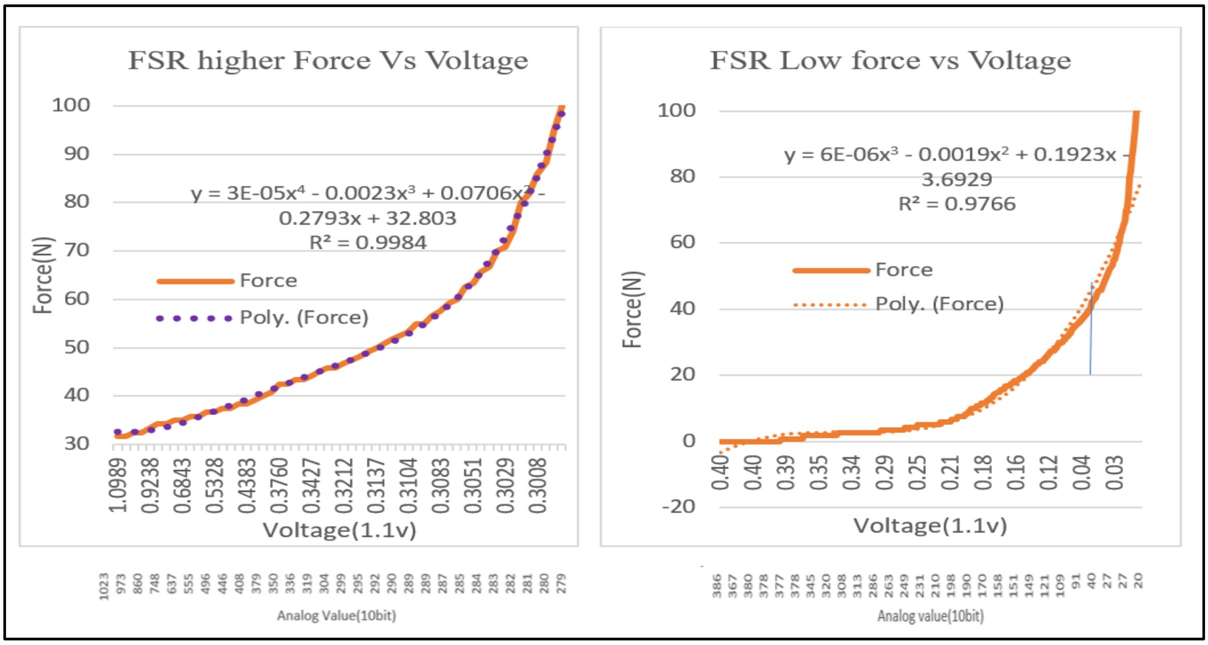

Figure S2. Calibration Curve for higher force (Leftside) to measure 30N to 100N and lower force (rightside) to measures 1N to 30N. After designing the circuit with Op-Amp, the FSR was calibrated with the materials testing machine.

2. <u>Distance Sensors calibration and accuracy testing</u>

An initial study found that 'IR-ToF sensors' (VL6180X, STMicroelectronics, Geneva, Switzerland) can provide better accuracy than alternative technologies (Bertuletti, Cereatti et al. 2016) [24]. Furthermore, it should be noticed that, in general, the sensor manufacturers (e.g., ST Microelectronics) only report in the datasheet the sensor performance under very specific and controlled conditions. Therefore, it is crucial to evaluate the system performance under working conditions simulating the real scenarios.

For this specific study, the IR-ToF proximity sensor sampling rate was set to 20 Hz, and, since the minimum foot clearance is generally less than 250 mm, so the measurement range was set to 0–250 mm.

We analysed the following factors, which could potentially affect the accuracy associated with distance estimation:

(i) Colours of the target surface (grey carpet, white carpet, and wood)

(ii) Distance (from 0 to 250 mm)

(iii) Angle of incidence (0,10,20,30, 40,50,60)

All three influencing factors were tested both in static and dynamic conditions. These factors were chosen to cover the range of possible configurations occurring during stair walking. In static acquisitions, the target (carpet) was kept stationary in front of the 'VL6180x distance sensor' (fitted in the shoe). While during dynamic acquisitions, the sensor shoe was moved to the desired position.

Supplementary Materials

The first distance experiment was done with all these three light grey carpets, dark grey carpet, and wood material. Distance sensors were fitted with shoes, and it was kept at 28mm from the wood, light grey and dark grey carpet. Similar results were found for all the materials tested. According to VL6180X distance sensor's datasheet, accuracy is ±3mm; this is clear from this experiment as well .Based on the results provided by the initial investigation on the influence of the colour of the target, we decided to use the light grey carpet for the subsequent experimental acquisitions.

The second experiment was done to check the accuracy of Angle of Incidence (AoI) and Distance measurement. The distance measurement was done whilst the AoI was kept from zero degrees to 60 degrees by increasing it in 10-degree (0^0, 10^0,20^o, 30°,40°,50°, 60°). For each testing, the 'mean value' of the distance provided by the sensor and actual distance (manually measured by the ruler) were computed, and the absolute difference of these two was derived.

The overall 'Mean error (BIAS)', 'Mean absolute error (MAE)' and 'Mean absolute percentage error (MAE%)' were computed by averaging differences over a few trials. The 'Root Mean Squared Error (RMSE)' was calculated.

Table S1 shows the calculation for distance sensor offset and accuracy testing for a different angle of incidence (AoI). Offsets and accuracy were calculated Using the above formulas. Our results showed that low BIAS rate (1mm) for this sensor and the absolute error for the sensor was 2.4mm, which was less than the maximum given by the company's sensor specifications. Also, the RMSE was only 7.85mm.

Table S1 Distance sensor offset and accuracy testing results.

Bias	1mm
MAE	2.4 mm
RMSE	7.85mm
MAPE	8.1%

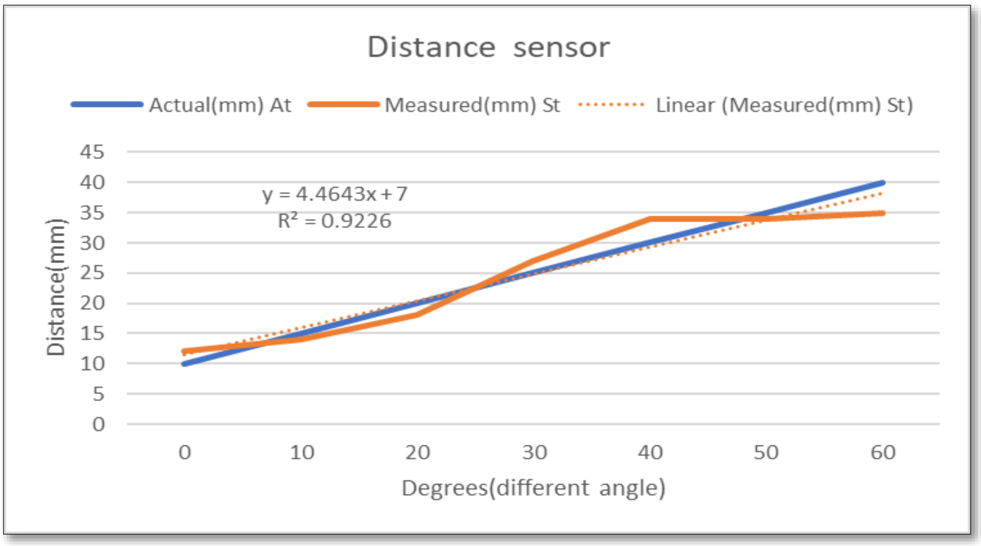

Figure S3. Distance sensor calibration curve, sensor angle set for zero degrees to 60 degrees by increasing it in 10° (0°, 10°, 20°, 30°, 40°, 50°, 60°), at the same distance was measured for 10 mm, 15 mm, 20 mm, 25 mm, 30 mm, 35 mm and 40 mm. Linear correlation $R^2 = 0.9226$.

Based on the above result, the calibration curve in Figure S3 shown in was plotted. The actual measurement and distance sensor measurement values formed a linear correlation. This Linear correlation calculated with the formula 'y= 4.4643x+7' to calibrate the range sensors and our 'R square value' was 0.9226, which confirmed a significant correlation between these two measurement values.
